# 1460. Assessing the impact of a surgical site infection prevention bundle on open reduction of fracture

**DOI:** 10.1093/ofid/ofad500.1297

**Published:** 2023-11-27

**Authors:** Raquel Bandeira da Silva, Braulio Couto, Thiago C Gontijo, Matheus Nascimento Otoni Vieira, Erik Alves, Rafael Silva Castro, Matheus Muniz, Sofia Mafra, Rafael Bandeira da Silva, Gabrielle Mota, Rodrigo Vieira, Glauco Messias, Mauro Salles

**Affiliations:** Hospital Universitário Ciências Médicas, Belo Horizonte, Minas Gerais, Brazil; Biobyte Tecnologia em Epidemiologia, Belo Horizonte, Minas Gerais, Brazil; University Hospital Medical Sciences, Belo Horizonte, Minas Gerais, Brazil; Hospital Universitário Ciências Médicas, Belo Horizonte, Minas Gerais, Brazil; Faculdade de Ciências Medicas de Minas Gerais, Belo Horizonte, Minas Gerais, Brazil; Hospital Universitário Ciências Médicas, Belo Horizonte, Minas Gerais, Brazil; Hospital Universitário Ciências Médicas (HUCM), Belo Horizonte, Minas Gerais, Brazil; Hospital Universitário Ciências Médicas (HUCM), Belo Horizonte, Minas Gerais, Brazil; Hospital Universitário Ciências Médicas (HUCM), Belo Horizonte, Minas Gerais, Brazil; Hospital Universitário Ciências Médicas (HUCM), Belo Horizonte, Minas Gerais, Brazil; Hospital Universitário Ciências Médicas (HUCM), Belo Horizonte, Minas Gerais, Brazil; Hospital Universitário Ciências Médicas (HUCM), Belo Horizonte, Minas Gerais, Brazil; Division of Infectious Diseases, Department of Internal Medicine, Santa Casa de Sao Paulo School of Medicine, Sao Paulo, Brazil., Sao Paulo, Sao Paulo, Brazil

## Abstract

**Background:**

In our hospital, risk of surgical site infection (SSI) after orthopaedic procedures varies approximately from 1% to 9% (Fig. 1). The objective of this study was to estimate the impact of a SSI prevention Bundle on the fracture-related infection (FRI) rates.

**Figure 1:**
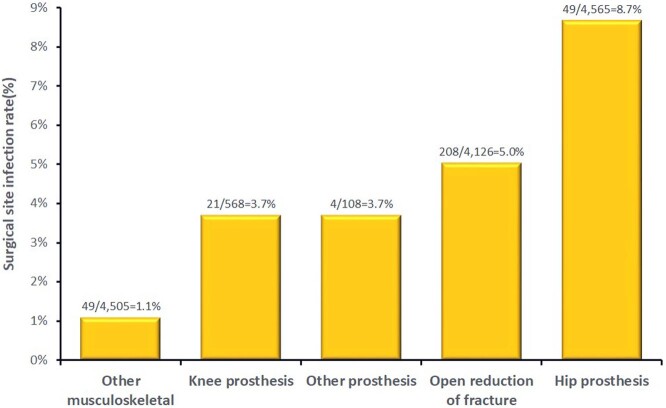
Surgical site infection (SSI) risk after orthopaedic procedures. Hospital Universitário Ciências Médicas (HUCM), Jan/2018 to Dec/2022.

**Methods:**

This is a single-center retrospective cohort study carried out between Jan/2021 and Dec/2022, which included patients undergoing any type of orthopaedic surgery for bone stabilization due to open fracture in a tertiary specialized public hospital in Belo Horizonte, a 3,000,000 inhabitants city from Brazil. Rates of SSI were assessed following implementing a Bundle that applied extended-spectrum antibiotics (cefuroxime with gentamicin) for prophylaxis towards patients at higher risk for postoperative SSI consisting of those with Charlson's comorbidity index score ≥ 5 and/or preoperative hospitalization longer than five days; intraoperative redosing of antibiotics for surgeries longer than 3 hours; carry out intraoperative rechecks, auditing surgical procedures using the infection prevention control team, and reinforcing good practices in the operating room (Fig. 2). The pre-intervention (Jan/2021 to Mar/2022) and post-intervention period (Apr/2022 to Dec/2022) were compared. Categorical variables were compared using the chi-square test, and continuous variables using the t-student test, considering a significance level of 5%. SSI risk and protective factors were identified by univariate, and multivariate analysis by logistic regression.

**Figure 2:**
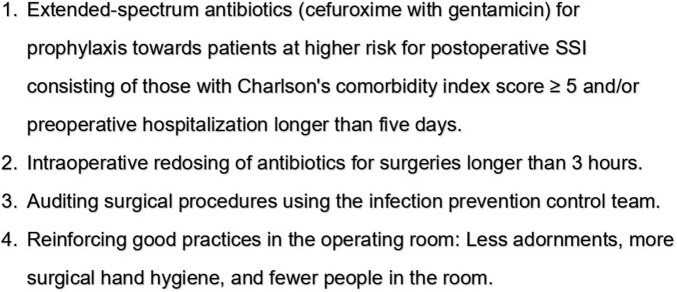
Surgical site infection prevention bundle. Hospital Universitário Ciências Médicas (HUCM), Apr/2022 to Dec/2022.

**Results:**

1,901 patients were included and analyzed: 1,037 and 864 were included in the “pre-intervention bundles” group and “post-intervention bundles” group, respectively. Rates of SSI in the “pre-intervention bundles” group and “post-intervention bundles” group were 6.9% (72/1037) and 2.8% (24/864), respectively (p<0.001). The overall risk for FRI following the intervention bundle dropped by 59% (Fig. 3). In a multivariate analysis, the relative risk of FRI after the Bundle implementation was 0.4 (Figs. 4, 5).

**Figure 3:**
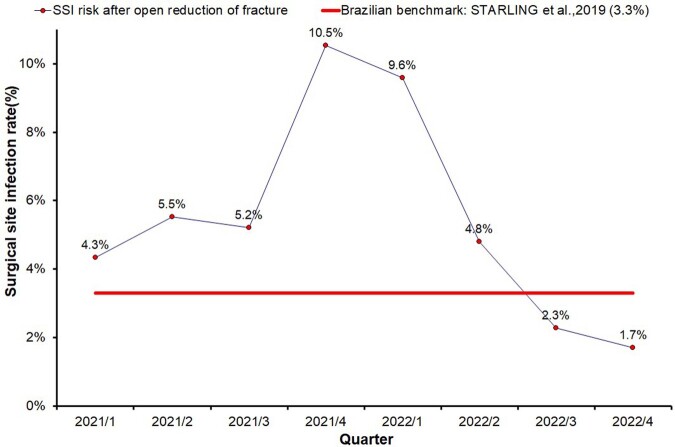
Quarterly SSI rate after open reduction of fracture. Hospital Universitário Ciências Médicas (HUCM), Jan/2021 to Dec/2022.

**Figure 4:**
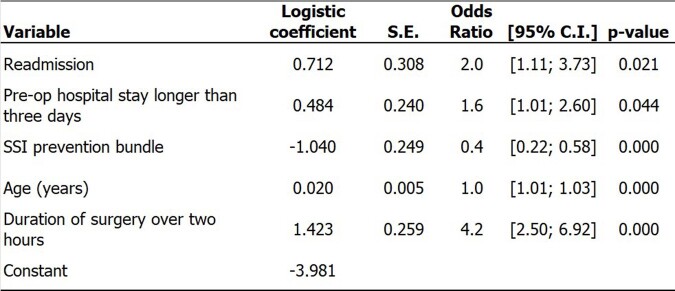
Logistic model for SSI after open reduction of fracture. Hospital Universitário Ciências Médicas (HUCM), Jan/2021 to Dec/2022.

**Figure 5:**
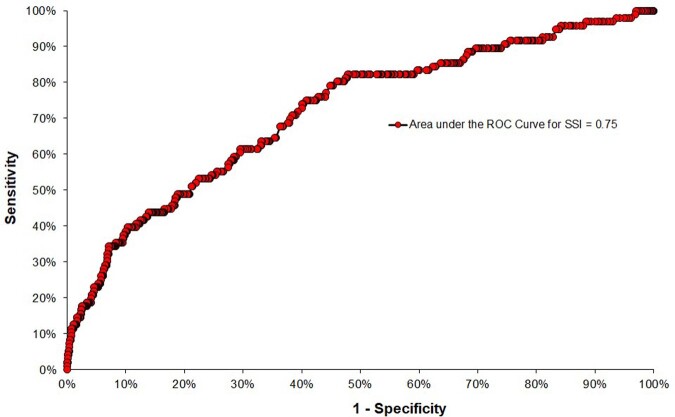
ROC curve for the logistic model for SSI after open reduction of fracture. Hospital Universitário Ciências Médicas (HUCM), Jan/2021 to Dec/2022.

**Conclusion:**

The Bundle strategy implemented was effective in reducing the rates of FRI. These simple and affordable measures are suitable to be applied and replicated in low-income specialized public orthopaedic centers.

**Disclosures:**

**All Authors**: No reported disclosures

